# Luobuma Leaf Spot Disease Caused by *Alternaria tenuissima* in China

**DOI:** 10.3390/jof9111062

**Published:** 2023-10-30

**Authors:** Yanru Lan, Zhichen Yan, Tingyu Duan

**Affiliations:** 1 State Key Laboratory of Herbage Improvement and Grassland Agro-Ecosystems, Lanzhou University, Lanzhou 730020, China; 2 Key Laboratory of Grassland Livestock Industry Innovation, Ministry of Agriculture and Rural Affairs, Lanzhou 730020, China; 3 College of Pastoral Agriculture Science and Technology, Lanzhou University, Lanzhou 730020, China

**Keywords:** *Alternaria tenuissima*, Luobuma, leaf spot disease, control

## Abstract

Luobuma (*Apocynum venetum* and *Poacynum hendersonni*) is widely cultivated for environmental conservation, medicinal purposes and the textile industry. In 2018, a severe leaf spot disease that attacked the leaves of Luobuma was observed in plants cultivated in Yuzhong County, Gansu Province, China. Symptoms of the disease appeared as white or off-white spots surrounded by brown margins on the leaves of *A. venetum*. The spots expanded and covered a large area of the leaf, presenting as “cankers” with progression of the disease, leading to leaf death. The initial symptoms of the disease on *P. hendersonni* were similar to the symptoms of *A. venetum*, with a larger disease spot than *A. venetum,* and the spot was black and thicker. The aim of this study was to identify the fungal species and evaluate the effectiveness of fungicides (hymexazol and zhongshengmycin) against the pathogen in vitro. The fungi species that caused the new disease was identified as *Alternaria tenuissima* based on the morphological characteristics, pathogenicity tests, and phylogenetic analysis of the internal transcribed spacer (*ITS*) region, glyceraldehyde 3-phosphate dehydrogenase (*gpd*), translation elongation factor 1-alpha (*TEF*) and the histone 3 (*H3*) gene sequences. The findings showed that hymexazol fungicide can be used to control leaf spot disease. This is the first report on Luobuma leaf spot disease caused by *A. tenuissima* in China.

## 1. Introduction

Luobuma (Apocynaceae) is a half-shrub or perennial herbaceous plant that grows in arid areas or dry periods as it is a phreatophyte [1]. This plant is widely distributed in central Asia and northwest China [2]. Luobuma species are divided into two genera comprising three species, *Apocynum venetum*, *Poacynum pictum* and *P. hendersonni* [3]. Fungal disease is the main factor that limits the growth and decreases the yield of Luobuma. In addition, fungal disease affects the color, appearance and taste of tea from Luobuma [4]. Rust disease caused by *Melampsora apocyni* is the most aggressive Luobuma foliar disease [4,5]. The second leading fungal disease in Luobuma is spot blight, reported in *A. venetum* and *P. pictum* species, which is caused by *Septoria apocyni* and is characterized circular to irregular lesions on leaves, resulting in leaf yellowing and the falling of leaves [6]. Other severe diseases reported in this plant include black leaf spot disease caused by *Alternaria catharanthicola*, and root rot caused by *Fusarium solani*. *Alternaria catharanthicola* causes the withering and drying of leaves and defoliation [7]. The main symptoms of *F. solani* root rot include wilting, darkening, and rot of the root collar and vascular bundle, which lead to defoliation and death of the plant [8]. In addition, a new disease known as *Boeremia exigua* that causes stem necrotic lesions on Luobuma was reported in 2022 [9].

*Alternaria* spp. are saprophytic fungi abundant in the environment, which are serious and potentially destructive pathogens causing invasive diseases to many hosts [10]. Different species of *Alternaria* can infect the same plant, causing different diseases. Eight *Alternaria* species (*A. alternata*, *A. tenuissima*, *A. dumosa*, *A. arborescens*, *A. infectoria*, *A. grandis*, *A. interrupra*, and *A. solani*) have been reported that cause potato (*Solanum tuberosum*) foliar disease, resulting in significant yield losses in potato farming globally [11]. Nine species of *Alternaria* (*A. helianthi*, *A. alternata*, *A. zinniae*, *A. tenuissima*, *A. leucanthemi*, *A. helianthicola*, *A. longissima*, *A. helianthinficiens*, and *A. protenta*) have been reported that infect sunflower (*Helianthus annuus*), causing leaf spot diseases [12]. *Alternaria catharanthicola* associated with *A. venetum* was reported in China in the recent past [7]. In the present study, we observed severe leaf spot disease associated with *Alternaria* on the leaves and stems of Luobuma (*A. venetum* and *P. hendersonni*) cultivated in Yuzhong, Gansu Province, China. The incidence of *A. venetum* and *P. hendersonni* were up to 77.78% and 95.56% between 2018 and 2019. 

Species delimitation in *Alternaria* species is challenging, especially the identification of small-spored *Alternaria* spp. The combination of morphological characterization with molecular traits is an effective strategy for the classification and identification of fungi. In addition, the use of accurate loci analysis is important for the identification of fungi species. Previous findings have showed that *A. alternata* and *A. tenuissima* generally cluster in one clade, based on the molecular analysis of *Alt a1* exon and *gpd* exon sequences [13]. However, subsequent studies showed that sequence analyses of the partial coding sequences of histone 3 gene effectively discriminated the two *Alternaria* species [12]. 

Chemical control plays an important role in integrated disease control, with advantages such as a rapid effect and simple application method, the ability to be used as emergency method, and the approach is not restricted by regional and seasonal effects. Hymexazol, with the chemical name 3-hydroxy-5-methylisoxazol, is a broad-spectrum fungicide used to control root rot diseases, especially soil-borne diseases caused by *Fusarium* [14], *Pythium* and *Aphanomyces* [15] on rice (*Oryza sativa*) or vegetables. Zhongshengmycin (ZSM) is a new agricultural antibiotic used for fungal disease control in plants, which is produced by *Streptomyces lavendulae* var. *hainanensisn* [16]. Fungicides containing ZSM are effective in controlling crop diseases, including bacterial and fungal plant diseases, such as leaf blight disease (*Pantoea agglomerans*) in oats (*Avena sativa*) [17], bacterial leaf blight (*Xanthomonas oryzae* pv. *oryzae*) in rice (*O. sativa*) [18] and canker disease (*Pseudomonas syringae* pv. *actinidiae*) that affects kiwifruit (*Actinidia*) [19]. Therefore, in the present study, we explored the effect of the two fungicides, hymexazol and ZSM, to control the disease caused by *Alternaria* sp. The objectives of the study were (1) to identify the pathogen that caused leaf spot disease on two Luobuma species, *A. venetum* and *P. hendersonn,* and (2) to explore an effective fungicide to control the disease. 

## 2. Materials and Methods

### 2.1. Disease Survey 

The Luobuma field was an experimental field comprising *Apocynum venetum* and *Poacynum hendersonni* that was established in 2017 in Yuzhong County, China using the seeds of these species [9]. In 2018, severe leaf spot was observed on the leaves of *A. venetum* and *P. hendersonii* species in the experimental field in Yuzhong, Gansu Province, China. We performed successive disease surveillance of the two species during the growth seasons from 2018 to 2019 to determine the incidence and disease index of leaf spot disease. The investigations were carried out on 29 September 2018, and 23 June and 23 September 2019. The disease surveillance was conducted based on a method used to investigate *A. venetum* rust [20]. The severity of leaf spot disease was visually estimated as the percentage of leaves covered with black scab and the results were expressed on a scale of 0–5 as follows: 0 for no signs of infection; 1 for 0.1% to 5% leaf area covered with black scab; 2 for 5.1% to 20%; 3 for 20.1% to 50%; 4 for 50.1% to 75%; and 5 for >75.1%. The disease incidence and disease index were determined using a method described by Lan et al. [9].

### 2.2. Sample Collection and Isolation

Symptomatic leaves were collected from the two-species evaluation of leaf spot symptoms and pathogen isolation analysis on 29 September 2018 and 23 June 2019. The symptomatic leaves were placed in an envelope and were stored at 4 °C for subsequent pathogen isolation. After 48 h, pathogens were isolated from 10 symptomatic leaves sampled from each species. Briefly, the diseased leaves were surface-sterilized using 75% ethanol for 45 s, then rinsed thrice with sterile distilled water and air-dried on sterile filter paper. The diseased spot margin was then cut and plated on a Petri dish containing potato dextrose agar (PDA). Two dishes were prepared from each leaf, and ten segments (~2 mm) were placed in each dish. Three strains were isolated from *A. venetum* (AvAt-1, AvAt-2, AvAt-3) and *P. hendersonii* (PhAt-1, PhAt-2, PhAt-3) then subjected to subsequent morphological and molecular identification. The AvAt-2 and PhAt-3 strains were used as representative strains for a pathogenicity test and fungicide virulence test.

### 2.3. Morphological and Biological Characterization 

The six strains isolated from *A. venetum* and *P. hendersonii* were purified and cultured for seven days at 25 ± 1 °C to explore the morphological characteristics and biological traits of *A. tenuissima*. Subsequently, the plugs of hyphae (4 mm in diameter) of each strain were removed from the edges of 7-day-old colonies and transferred to the center of a new Petri dish containing PDA substrate, then incubated at 25 ± 1 °C under a 14:10 h light–dark cycle. Four replicates were conducted for each strain. After seven days, conidia and conidiophores were examined using light microscopy (Olympus BX51, Tokyo, Japan). The sizes of 100 conidia for each strain were measured under the microscope. Morphospecies assignments were assigned based on descriptions of *Alternaria* spp. by Simmons [21]. 

The effect of different culture medium, temperature, pH, carbon source and nitrogen source on the biological characteristics of *A. tenuissima* was determined. Different culture media, including PDA, potato carrot agar (PCA), potato saccharose agar (PSA), Czapek dox agar (CDA), and Luobuma agar (LA) (the fresh leaves of *Apocynum venetum* at 20 g/L and agar at 18 g/L) were evaluated. To assess the effect of varying temperatures on the morphology of *A. tenuissima*, the PDA medium was used to culture the mycelial plugs (4 mm diameter) at a temperature gradient of 5 °C, 10 °C, 15 °C, 20 °C, 25 °C, 30 °C, and 35 °C. The pH value of medium containing 15 mL PDA was adjusted to 5, 6, 7, 8, 9, 10 and 11 to assess the effect of varying pH on the morphology of *A. tenuissima*. We assessed the effect of nitrogen and carbon sources on *A. tenuissima* by adding starch, fructose, sucrose, glucose, NH_4_NO_3_, KNO_3_, NaNO_3_ and beef extract to the PDA medium. Four replicates were conducted for each medium substrate. Colony diameter was measured using the criss-cross method after culturing for six days to determine a daily growth rate of *A. tenuissima* [22].

### 2.4. DNA Extraction, Polymerase Chain Reaction (PCR), Sequencing and Phylogenetic Analysis 

Total genomic DNA was extracted from 30 mg of fungal pathogens using the Fungal Mini Kit (Omega bio-Tek, Doraville, CA, USA) according to the manufacturer’s protocol with little modifications. The purity and concentration of the total DNA were assessed using a NanoDrop 1000 spectrophotometer (NanoDrop Technologies, Wilmington, DE, USA). The DNA was stored at −20 °C for subsequent analysis. Amplification was performed with *ITS1* (5′-TCCGTAGGTGAACCTGCGG-3′) and *ITS4* (5′- TCCTCCGCTTATTGATATGC-3′) primers [23]. PCR reactions were carried out using a DLAB PCR TC1000-G thermocycler (Beijing, China) with a final volume of 25 μL, which comprised 9 μL of ddH_2_O, 1 μL of each primer (forward and reverse), 1.5 μL of template DNA, and 12.5 μL of DNA polymerase (2X SanTaq Fast PCR Master Mix with Blue Dye). The PCR amplification process comprised an initial denaturation at 95 °C for 5 min, 35 cycles of denaturation at 95 °C for 30 s, annealing at 53 °C for 2 min, extension at 72 °C for 30 s, and final extension at 72 °C for 8 min. A negative control (ddH_2_O instead of template DNA) was included in all experiments. The effectiveness of PCR and the length of the amplified DNA fragments were evaluated by conducting 1.5% agarose gel electrophoresis. DNA sequencing was performed by Sangon Biotech (Wuhan, China). Amplification of glyceraldehyde 3-phosphate dehydrogenase (*gpd*) was performed with *gpd1* (5′-CAACGGCTTCGGTCGCATTG-3′) and *gpd2* (5′-GCCAAGCAGTTGGTTGTGC-3′) primers [24]. Amplification of translation elongation factor *1-alpha (TEF*) was performed with *EF1-728F* (5′-CATCGAGAAGTTCGAGAAGG-3′) and *EF1-986R* (5′-TACTTGAAGGAACCCTTACC -3′) primers [25]. The histone 3 gene was amplified using *H3-1a* (5′-ACTAAGCAGACCGCCCGCAGG-3′) and *H3-1b* (5′-GCGGGCGAGCTGGATGTCCTT-3′) primers, which is sufficient at discriminating between *A. tenuissima* and *A. alternata* [12]. The PCR reactions were conducted following the same experimental procedure used for the *ITS* region apart from annealing *gpd1/2* primers at 54 °C for 2 min, annealing *TEF* primers at 52 °C for 2 min, and annealing *H3* primers at 55 °C for 2 min. Sequence contigs were assembled using DNAbaser software and the sequences were deposited in the GenBank database. Sequences for the three target regions, *ITS*, *gpd*, *TEF* and *H3*, were used for phylogenetic analysis using MEGA version 7 with the maximum likelihood (ML) method [26].

### 2.5. Pathogenicity Test

The AvAt-2 and PhAt-3 strains and the two Luobuma species were used for the pathogenicity test. *A. venetum* and *P. hendersonni* seeds were collected from Altay, Xinjiang. The seeds were removed from the pods and sterilized with 75% alcohol before germination, and then they were plated in Petri dishes with two layers of sterilized filter paper and 5 mL sterile distilled water. Vermiculite was sterilized twice for 3 h at 121 °C in an autoclave. The seedlings were transplanted to 15 cm diameter pots containing sterilized vermiculite. Five seedlings were planted in each pot. Plants were grown under artificial light to provide a 4 h photoperiod. The average greenhouse temperature was 25 °C during the day and 17 °C at night, and the average relative humidity (RH) was 65% during the day and 78% at night. 

The two strains were cultured for seven days on the PDA plates for inoculum preparation. Spores with hypha of the two strains were transferred to a beaker containing 100 mL sterile water and two drops of Tween 80 to achieve spore suspension. The spore suspension was filtered using two layers of sterilized gauze until 1 × 10^7^ conidia/mL spore suspensions with little hypha were obtained. The inoculum of the two strains was separately inoculated in *A. venetum* and *P. hendersonni*. Three months after transplanting, fifteen pots for each species were divided into three groups, with five pots in each group. The plants were inoculated with spore suspension (1 × 10^7^ conidia/mL) of AvAt-2 strain, spore suspension (1 × 10^7^ conidia/mL) of PhAt-3 strain, and sterile distilled water with five pots for each treatment. The plants were covered with a black plastic bag for 48 h after inoculation with the fungus.

### 2.6. Fungicide Virulence Test

The virulence of two fungicides, namely hymexazol (a.i. hymexazol 70%, Shandong, China) and ZSM (a.i. ZSM 2.5% + amino-oligosaccharin 7.5%, Beijing, China) against AvAt-2 and PhAt-3 strains were evaluated. Different concentration gradients of hymexazol (58, 117, 233, 467, and 933 mg/L) and ZSM (17, 33, 67, 133, and 267 mg/L) were prepared and added to sterilized PDA media. PDA medium with no fungicides was used as the control treatment. The hyphae plugs (4 mm in diameter) of the two strains were collected from the edges of seven-day-old colonies on PDA and transferred to the center of the Petri dish containing different concentration fungicides, and incubated at 25 ± 1 °C under a 14:10 h light–dark cycle. The colony diameter was measured using the criss-cross method after six days [22]. 

## 3. Results

### 3.1. Symptoms of Leaf Spot Disease

**Figure 1:**
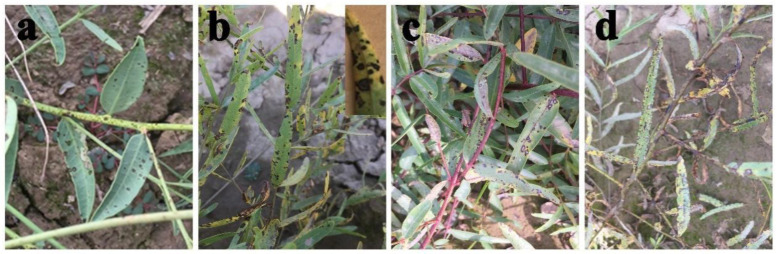
The symptom of Luobuma leaf spot disease caused by *A. tenuissima* in the field. (**a**,**b**): early and late stages of *P. hendersonni* leaf spot disease; (**c**,**d**): early and late stages of *A. tenuissima* leaf spot disease.

A foliar disease caused by the pathogen *Alternaria* sp. was observed on *A. venetum* and *P. hendersonni* plants cultivated in Yuzhong County, Gansu Province, China, between 2018 and 2019. The symptoms of the two species exhibited slight differences. The initial symptoms included approximately 1 mm small circular spot with a dark brown margin and white or off-white color in the center of disease spots, which appeared on leaves and shoots of *P. hendersonni* plants in late May. The disease spots became black and thick with an increase in disease spot size. The boundary between diseased and healthy leaves was clearly defined throughout the disease process. The initial symptoms of leaf spot disease on the *A. venetum* plants were similar to the symptoms on *P. hendersonni* plants, but the disease spots in *A. venetum* were characterized by a reddish-brown margin. The spots became dark brown, gradually enlarged and eventually coalesced upon disease progression. The spots turned white, exhibited a depression, had a brown margin, and the boundary between the diseased and healthy areas were evident. Under severe disease conditions, the whole leaf was covered with white spots and exhibited “canker” symptoms, which causes extensive leaf senescence (Figure 1).

### 3.2. Disease Survey

**Figure 2:**
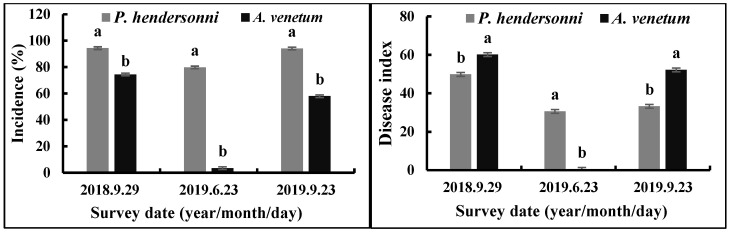
Incidence and disease indexes of leaf spot disease caused by *A. tenuissima* on *P. hendersonni* and *A. venetum* plants cultivated in the field between 2018 and 2019. Notes: different lowercase letters indicate significant differences between *P. hendersonni* and *A. venetum* on same date at *p* < 0.05.

Between 2018 and 2019, the incidence of *P. hendersonni* leaf spot disease was significantly (*p* < 0.05) higher than *A. venetum* leaf spot disease. The disease was found firstly in 29 September 2018; the incidence of the disease of *P. hendersonni* and *A. venetum* were, respectively, 94.33% and 74.43%, with the disease index being 49.84 and 60.13. In 2019, the outbreak of the *Alternaria* sp. infecting *P. hendersonni* was earlier than the *A. venetum* plant. The pathogen infecting *P. hendersonni* began to outbreak in middle May, while the one infecting *A. venetum* was in the middle of June. On the 23rd June, the incidence and disease index of *P. hendersonni* reached 79.73% and 30.58, respectively. The incidence and disease index of *A. venetum* were slight, with 3.46% and 0.36, respectively (Figure 2). 

### 3.3. Morphological Characteristics of A. tenuissima 

**Figure 3:**
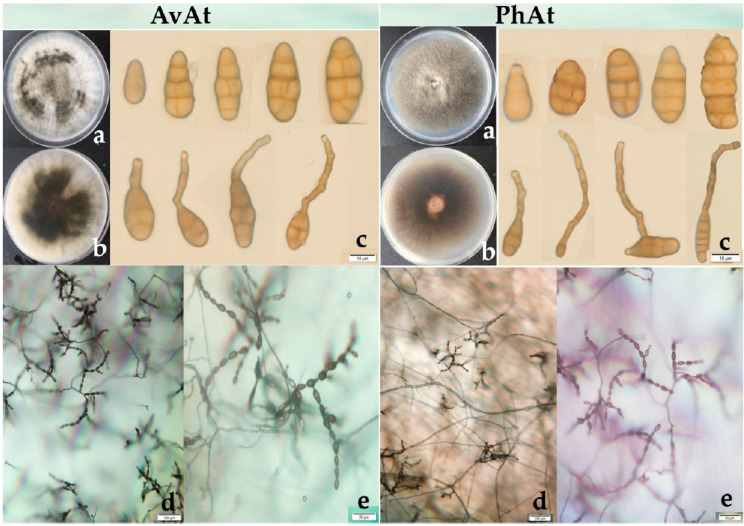
(**a**,**b**): colony of *A. tenuissima* (strains AvAt-2 and PhAt-3); (**c**): conidia of *A. tenuissima*; (**d**,**e**): the sporulation patterns of *A. tenuissima*.

In total, 48 strains of *Alternaria* species were isolated from infected leaves of the two plants in 2018 and 2019. The average isolation frequency of the pathogen believed to be *Alternaria* spp. from symptomatic leaves of *A. venetum* and *P. hendersonii* was 73.98% and 68.22%, respectively. The 48 strains were grouped into two types based on the morphology of the colony, as shown in Figure 3. Two types of *Alternaria* species were all observed in the strains isolated from symptomatic leaves of *A. venetum* and *P. hendersonii*. One type exhibited abundant aerial hyphae, with loose cottony and heterogenous greyish-green to olive-brown colonies on PDA plates. Most of the strains for the first type were isolated from the diseased leaves of *A. venetum* and were named the AvAt group. The other strains exhibited relatively sparse aerial mycelia, with homogenous greyish-green to olive-brown colonies on PDA plates. Most of these strains were isolated from the diseased leaves of *P. hendersonii* and were named the PhAt group. 

Six strains from leaf spot disease on *A. venetum* (AvAt-1, AvAt-2, and AvAt-3) and *P. hendersonni* (PhAt-1, PhAt-2, and PhAt-3) were sampled randomly for biological characterization. The strains were incubated on PDA for seven days and the colonies of the AvAt and PhAt groups had an average diameter of 68.50 mm and 70.20 mm, respectively. The AvAt and PhAt groups had an average diameter of 70.25 mm and 73.55 mm on PCA, respectively. The conidia of the two groups were 15.31 − 47.18 × 7.65 − 12.69 µm (average 9.75 μm × 31.07 μm) and exhibited ovoid to obclavate shapes. The number of transverse septa and longitudinal septa of conidia in the AvAt and PhAt strains varied from 1 to 5 and from 0 to 2, respectively. Unbranched conidial chains with up to 14 conidia in length were observed on the strains cultured on PDA. 

### 3.4. Biological Characteristics

The six representative strains from AvAt and PhAt groups exhibited a large colon diameter after culturing on OA and PSA media, but CDA media was not conducive for the growth of those strains. In addition, those strains grow effectively under an alkaline nutritional environment. The optimum temperature, carbon source, and nitrogen source to the growth of the six representative strains were 25 °C, glucose, and beef extract, respectively (Table 1). 

The results showed that the growth rate of the PhAt strains isolated from *P. hendersonni* was significantly (*p* < 0.05) higher than AvAt strains isolated from *A. venetum* when the representative strains were cultured on LA or in alkaline or acidity nutritional environment or 5 °C, or on NH_4_NO_3_ or starch carbon source conditions. However, when the two types of strains were cultured at 35 °C, or on beef extract nitrogen source conditions, the growth rate of AvAt strains isolated from *A. venetum* was significantly (*p* < 0.05) higher than PhAt strains isolated from *P. hendersonni* (Table 1).

### 3.5. DNA Sequencing and Phylogenetic Analysis

PCR amplification of the ITS region of rDNA from the six strains generated 531 bp fragments. Blast searches showed that rDNA *ITS* sequences of the *Alternaria* isolates were 100% similar to sequences from *A. alternata* (GenBank accession no. MT453271.1), *A. tenuissima* (KJ082100.1) and several other *Alternaria* species. PCR amplification of the *gpd* region of rDNA from the six strains generated 595 bp fragments. Blast searches showed that rDNA *gpd* sequences of the *Alternaria* isolates were 100% similar to sequences from *A. alternata* (GenBank accession no. MH047224.1). Blast searches showed that rDNA *TEF* sequences of the *Alternaria* isolates were 100% similar to *A. tenuissima* sequences (GenBank accession no. OR485421.1). However, the BLAST search of the partial coding sequence of the histone 3 gene revealed that the six strains were >99% similar to *A. tenuissima*, and 504 bp fragments were obtained for this gene.

**Figure 4:**
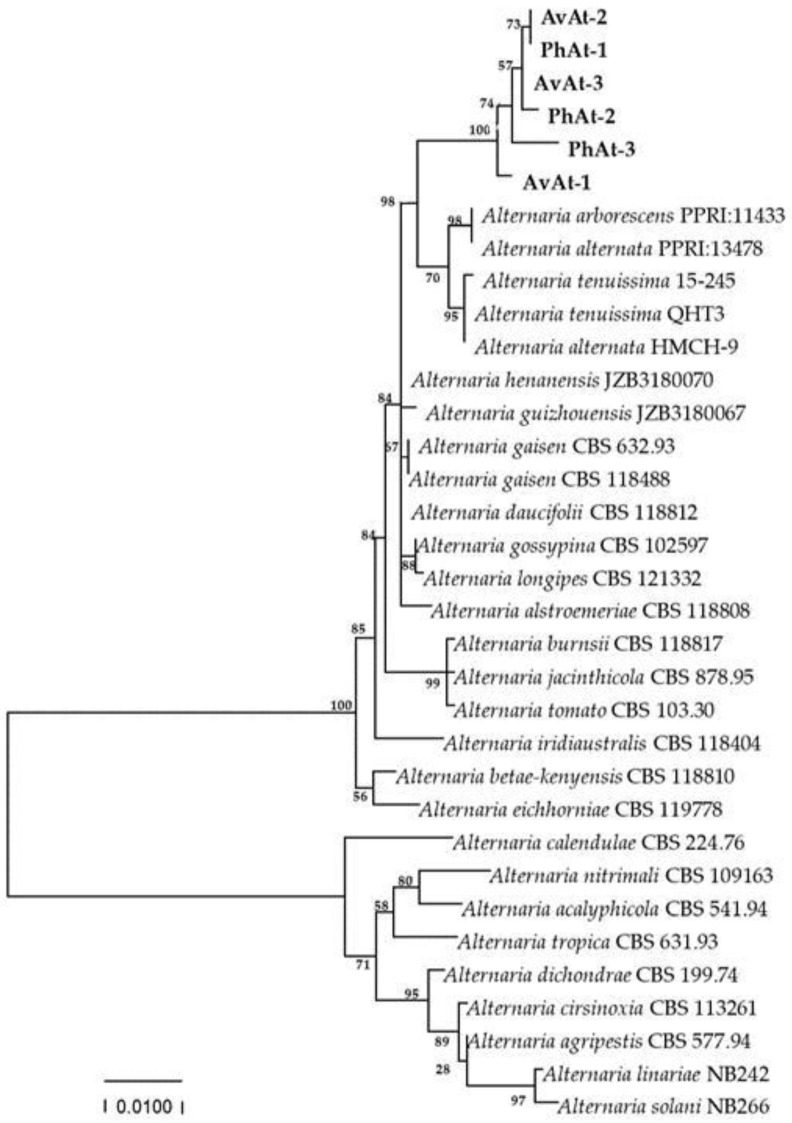
Phylogenetic tree obtained from Maximum Likelihood analysis with Tamura–Nei model based on the combined gene sequences of *ITS*, *gpd*, and *tTEF* sequences from *Alternaria* spp. Bootstrap values (1000 replicates) above were shown at the nodes. Strain numbers were listed in next to the species. Strains obtained in this study were shown in bold.

**Figure 5:**
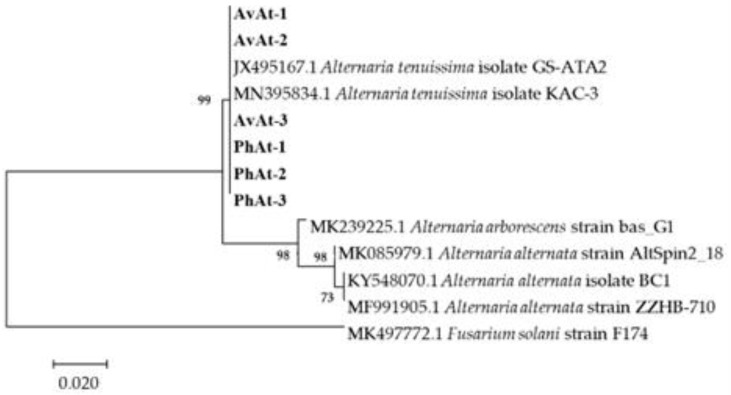
Phylogenetic tree obtained from Maximum Likelihood analysis with Tamura–Nei model based on *H3* sequences. Bootstrap values (1000 replicates) above are shown at the nodes. Strain numbers are listed in next to the species. Strains obtained in this study are shown in bold. *Fusarium solani* (MK497772.1) is used as an outgroup.

Phylogenetic trees were constructed based on the sequences of the rDNA *ITS*, *gpd*, and *TEF* regions of 25 *Alternaria* species obtained by blasting in GENBANK (Table 2). The phylogenetic results revealed that the strains in our study were close to *A. arborescens*, *A. alternata* and *A. tenuissima* clusters (Figure 4). Phylogenetic analysis based on the partial coding sequence of the histone 3 gene showed that the strains isolated in this study clustered in a individual *A. tenuissima* clade (Figure 5). Therefore, phylogenetic analysis was consistent with the Blast results, and combined morphological characteristics, the strains causing leaf spot disease of *A. venetum* and *P. hendersonni* was identified to *A. tenuissima*.

### 3.6. Pathogenicity Testing

**Figure 6:**
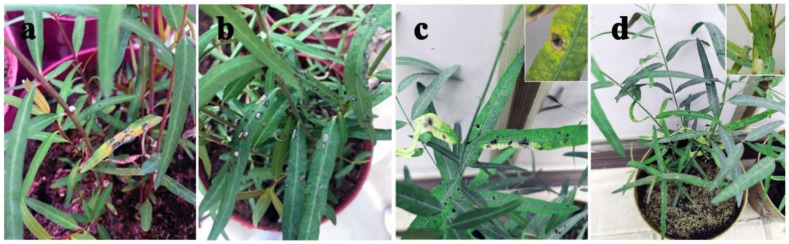
The symptoms of Luobuma leaf spot disease after 5 days inoculation with *A. tenuissima*. (**a**,**b**): leaf symptoms of *P. hendersonni* inoculated with AvAt and PhAt strain, respectively. (**c**,**d**): leaf symptoms of *A. venetum* inoculated with AvAt and PhAt strains.

Spore suspensions with 1 × 10^7^ conidia/mL of the two representative *A. tenuissima* (strains AvAt-2 and PhAt-3) were inoculated on *A. venetum* and *P. hendersonni* leaves to confirm Koch’s postulates. The two strains shown pathogenic to both of *A. venetum and P. hendersonni*, and severity was found in their host of origin. Three days after inoculation, the inoculated *A. venetum* and *P. hendersonni* leaves developed nearly round or oval discolored diseased spots, or dark brown to black lesions, slightly similar to the initial symptoms observed on the naturally infected leaf samples in the field (Figure 6). All control leaves inoculated with sterile distilled water remained asymptomatic and no *Alternaria* spp. was isolated from them. *Alternaria* species were re-isolated consistently from all the symptomatic leaves with lesion spots and species identities confirmed Koch’s postulates. The two strain groups of *Alternaria* species were all isolated from symptomatic Luobuma leaves inoculation with AvAt -2 and PhAt-3, respectively. 

### 3.7. Fungicide Virulence Test

**Figure 7:**
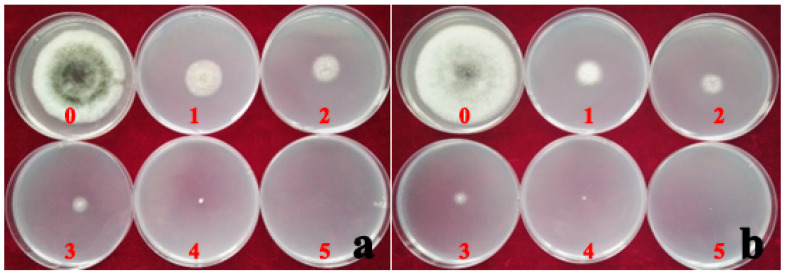
The inhibition rate of mycelium growth by the different concentrations of hymexazol fungicide on strain AvAt (**a**) and PhAt (**b**). Labels: 0: no hymexazol fungicide; 1, 2, 3, 4 and 5 indicate the results for the addition of 58, 117, 233, 467, and 988 mg/L hymexazol fungicide added to the PDA medium, respectively.

**Figure 8:**
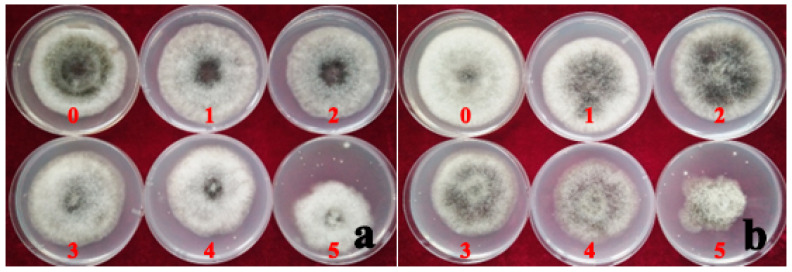
The inhibition rate of mycelium growth by the different concentrations of ZSM fungicide on strain AvAt (**a**) and PhAt (**b**). Labels: 0: No ZSM fungicide; 1, 2, 3, 4 and 5 indicate the results for the addition of 17, 33, 67, 133, and 267 mg/L ZSM fungicide added to the PDA medium, respectively.

The median effective concentration (EC50) of hymexazol fungicide was 84.64% less than the EC50 of ZSM fungicide. This implies that *A. tenuissima* had a relatively higher sensitivity to the hymexazol fungicide, and the PhAt strain was more sensitive to the fungicide than the AvAt strain (Table 3 and Table 4). The inhibition rate of mycelium growth increased gradually with an increase in the concentration of hymexazol fungicide in the PDA culture (Table 4; Figure 7). On the contrary, the rate of mycelium growth without increased with an increase in the concentration of ZSM fungicide in the PDA culture (Table 4; Figure 8). The inhibition rate of the mycelium growth of strains AvAt and PhAt were 88.10% and 94.08%, respectively, when the concentration of hymexazol was 467 mg/L (Table 4). These results show that hymexazol fungicide is effective in controlling leaf spot disease on Luobuma caused by *A. tenuissima* in China.

## 4. Discussion

In the present study, a severe foliar disease caused by *A. tenuissima* was observed in Luobuma in China in 2018. Numerous reports have demonstrated the saprophytic and pathogenic nature of *A. tenuissim*. Approximately 760 species of *Alternaria* have been listed on the Index Fungorum (www.indexfungorum.org, accessed on 18 Semptember 2020) database, with more than 300 confirmed species. *A. tenuissim* is a pathogenic fungus that infects several plants. This fungus causes leaf spot disease in *Capsicum annuum* [42], *Paris polyphylla* var. *chinensis* [43], *Solanum melongena* [44], *Farfugium japonicum* [45], *Triticum turgidum* [46], *Avicennia marina* [47], *Psidium guajava* [48], and *Aronia melanocarpa* [32]. Most of the symptoms of *A. tenuissima* infection include small circular or irregular brown leaf spots all over the foliage. At the later stages of infection, the spots gradually increase in size and eventually coalesce, resulting in withering, extensive drying and leaf senescence [32,42,43,44]. The disease spots of some hosts have unclear or clear concentric rings or zones [42]. More than eight and nine *Alternaria* species infect potato (*Solanum tuberosum*) and sunflower (*Helianthus annuus*), respectively, causing different diseases [11,12]. In the current study, a novel and severe leaf spot disease caused by *A. tenuissima* was observed on *A. venetum* and *P. hendersonni* in Yuzhong, Gansu province, China. The maximum disease incidence was 94.33% and 74.43% in *A. venetum* and *P. hendersonni* plants, respectively. A previous study reported *A. catharanthicola* infection on *A. venetum* in China [7]. The symptoms of the *A. tenuissima* infection on *A. venetum* and *P. hendersonni* presented differences. The visible slight differences in symptoms between *A. venetum* and *P. hendersonni* were associated with the stem color of the different ecotypes [49]. 

The species level delimitation of *Alternaria* is challenging due to lack of morphological characteristics and high morphological plasticity [50]. Accurate identification of the pathogen is important for effective disease management. In the present study, a preliminary classification was achieved based on the morphological characteristics. The pathogen causing leaf spot disease on Luobuma plants was identified as small-spored *Alternaria* species. The species-level classification of *Alternaria* was previously conducted by a combined phylogeny analysis of *ITS*, *TEF* and RNA polymerase II second-largest subunit gene (*RPB2*) [43] or using phylogenetic analysis of the individual genes [51,52]. However, the classification results of the same pathogen do not show strict congruence between morphology and phylogenetic lineage. *A. alternata* and *A. tenuissima* are morphologically distinct fungi, which cluster in one clade at the molecular level based on various gene locus such as *Alt a 1* exon, *gpd* exon sequences, and the endopolygalacturonase (*endoPG*) gene [13,51]. Therefore, a comprehensive combination of morphological and molecular methods is necessary for the identification of the small-spored *Alternaria* taxa that infect Luobuma in China. 

In this study, we used four genes (*ITS*, *gpd*, *TEF* and *H3*) to assess the classification of the pathogens causing Luobuma leaf spot disease. The phylogenetic tree based on three genes showed that the six strains clustered close to *A. arborescens*, *A. alternata* and *A. tenuissima*. The *ITS* region of rDNA of *A. catharanthicola*, which causes black leaf spot disease in *A. venetum* in China, was only retrieved from GENBANK. Therefore, it was distinguished based on its morphological characters, including the presence of single conidia or the formation of short branched chains (less than 10), and the presence of 3 to 8 transverse septa and 0 to 10 longitudinal and oblique septa [7]. A previous study conducted sequence analyses of the partial coding sequences of the histone 3 gene and the results showed that this approach accurately discriminated the two *Alternaria* species [12]. *Alternaria tenuissima* produces conidia as long (19.0 to 50.8, mean 34.4 µm) and unbranched chains of 7 to 20 elements, with one to six transverse septa and zero to three longitudinal septa [52,53]. Therefore, the strains that infected the leaves and stems of three Luobuma species were identified as *A. tenuissima* based on the molecular and biological data obtained in this study. 

The AvAt-2 and PhAt-3 strains of *A. tenuissima* identified in this study showed differences in colony, biological characteristics, pathogenicity, and fungicide virulence, but no significant differences were observed in other morphological characteristics such as conidia morphology and sporulation pattern. The PhAt-3 strain isolated from the diseased tissues of *P. hendersonni* could be adapted to a changing environment, such as adaptation to the different substrates, and a strong tolerance to a hostile environment (extreme temperatures or acidic or alkaline). But the PhAt-3 strain was more sensitive than the AvAt-2 strain to the hymexazol fungicide. This could be one of the factors for earlier and more serious occurrence of the *P. hendersonni* leaf spot disease. The pathogenicity testing indicated that severity was found in their host of origin. It indicated that the strains of *A. tenuissima* have host specificity. At a late stage of plant growth, the disease index of *P. hendersonni* leaf spot disease is lower than *A. venetum*. This phenomenon was related to the symptom that the spot was black and thick. Therefore, we suppose *P. hendersonni* is more resistant than *A. venetum* against *A. tenuissima*. At the end of the 2019 growing season, the growth of *P. hendersonni* was severely restricted by the disease. *P. hendersonni* did not grow in 2020, due to the attack of the disease in 2019. And the leaf spot disease caused by *A. tenuissima* is not also observed on the leaves of *A. venetum*. Therefore, the *P. hendersonni* could be alternative host of *A. tenuissima*.

Hymexazol is a common commercial fungicide with high antifungal activity against several fungal pathogens. This fungicide is commonly used as a positive control to assess the efficacy of other fungicides with novel structures to address the menace of resistance to existing fungicides. Hymexazol presents high antifungal activity against *A. solani* [54,55], *A. alternata* [56] and *A. tenuis* [57]. Hymexazol exhibited a 34.4 mg/L EC50 value against *A. alternata* and a 48% inhibition ratio to *A. alternata* [56]. A previous study reported that the inhibition rate of hymexazol against *A. tenuis* was 98.33% in vitro [57]. However, studies have not been conducted to explore the effect of hymexazol against *A. tenuissima*. ZSM controls most plant diseases caused by fungal and bacterial pathogens. ZSM inhibits the growth of the hyphae of *Didymella segeticola*, which causes tea (*Camellia sinensis*) leaf spot in vitro with a 5.9 µg/mL EC50 value [58] and *X. oryzae* pv. *oryzicola*, which causes rice bacterial blight disease [59,60]. In this study, the antifungal activity of ZSM against *A. tenuissima* was evaluated using the mycelium growth rate method. The results showed that ZSM was less effective in inhibiting the mycelial growth of *A. tenuissima*. In a previous report, agro-antibiotic ZSM had a weak inhibition effect against *Diaporthe eres*, the causal agent of *Polygonatum sibiricum* leaf blight [61]. These findings show that the accurate identification of the type of pathogen is important for disease management and control. In our study, the inhibition ratio of hymexazol was higher than ZSM against *A. tenuissima*, and hymexazol had a relatively lower EC50 value (151.75 mg/L), but it did not show excellent fungicidal effect. Rotation of ZSM and other fungicides can have synergistic and additive roles in controlling the Luobuma leaf spot disease. 

## 5. Conclusions

To the best of our knowledge, this is the first report of *A. tenuissima* causing leaf spot disease on Luobuma in China. The disease incidence in the field was approximately 90%, and the disease exhibited high incidence and severity. Hymexazol fungicide is effective in controlling Luobuma leaf spot disease caused by *A. tenuissima* in China. The present study provides useful information on pathogen identification, pathogenicity, and the occurrence of the leaf spot disease. This disease causes severe damage to Luoboma plants, so further research is required to explore strategies to control the disease effectively.

## Figures and Tables

**Table 1:** Growth rate (mm/6 d) of the representative strains isolated from the diseased *A. venetum* and *P. hendersonni* cultured on different mediums, carbon sources, nitrogen sources, pH values and temperatures.

Growth Conditions		AvAt-2	PhAt-3
	LA	50.75 ± 0.32 C b	57.00 ± 0.46 C a
	CDA	43.25 ± 0.48 D a	47.88 ± 0.22 D a
Culture media	OA	68.25 ± 1.89 A a	72.88 ± 0.43 A a
	PSA	67.00 ± 0.54 A a	69.88 ± 1.09 A a
	PDA	62.63 ± 0.80 B a	63.00 ± 0.35 B a
	Starch	54.13 ± 1.30 B b	58.50 ± 0.54 A a
	Fructose	53.37 ± 1.86 B a	51.50 ± 0.20 B a
Carbon source	Sucrose	52.63 ± 1.32 B a	51.37 ± 0.20 B a
	Glucose	60.50 ± 0.96 A a	61.00 ± 1.34 A a
	NaCl	44.75 ± 0.83 C b	49.38 ± 0.97 B a
	NH_4_NO_3_	39.63 ± 1.07 C b	45.00 ± 0.35 D a
	KNO_3_	53.25 ± 0.43 B a	53.50 ± 0.46 B a
Nitrogen source	NaNO_3_	51.50 ± 0.50 B a	51.00 ± 0.89 C a
	Beef extract	69.25 ± 0.32 A a	65.38 ± 0.55 A b
	NaCl	53.50 ± 0.74 B a	54.63 ± 0.38 B a
	5	30.63 ± 1.07 D b	37.50 ± 0.41 F a
	6	50.25 ± 0.32 C a	50.88 ± 0.77 E a
	7	56.75 ± 0.25 B a	56.25 ± 0.52 D a
pH values	8	57.38 ± 0.24 B b	59.13 ± 0.13 C a
	9	57.38 ± 0.38 B b	59.50 ± 0.46 C a
	10	58.00 ± 0.35 B b	61.75 ± 0.32 B a
	11	70.63 ± 0.55 A b	74.00 ± 0.20 A a
	5	16.10 ± 0.14 G b	17.30 ± 0.17 F a
	10	29.00 ± 0.90 F a	30.80 ± 0.69 E a
	15	52.10 ± 0.31 D a	53.30 ± 1.05 D a
Temperatures (℃)	20	81.20 ± 0.50 B a	82.20 ± 0.89 B a
	25	85.60 ± 0.95 A a	86.00 ± 1.15 A a
	30	72.20 ± 0.92 C a	72.60 ± 1.58 C a
	35	34.40 ± 0.56 E a	31.20 ± 0.53 E b

Notes: Different capital letters indicate significant differences within the same columns of each growth condition at *p* < 0.05; different lowercase letters indicate significant differences within the same rows at *p* < 0.05.

**Table 2:** Strain numbers, hosts and GenBank accessions from internal transcribed spacer (*ITS*), glyceraldehyde 3-phosphate dehydrogenase (*gpd*)*,* translation elongation factor 1-alpha (*tEF*), and histone H3 (*H3*) region of rDNA of the selected strains of *Alternaria* species were indicated in this study.

Species	Strains	Hosts/Substrates	Genbank Accessions				Literature
			*ITS*	*gpd*	*TEF*	*H3*	
*A. arborescens*	PPRI:11433	*Helianthus annuus*	MF381794	MF381768	MF381820	-	[27]
	bas_G1	*-*	-	-	-	MK239225	**-**
*A. alternata*	PPRI:13478	*H. annuus*	MF381802	MF381776	MF381828	-	[27]
	HMCH-9	*Lolium multiflorum*	MH567106	MH567107	MH567109	-	[28,29]
	BC1	*Brassica carinata*	-	-	-	KY548070	[29]
	AltSpin2_18	*Spinacia oleracea*	-	-	-	MK085979	[30]
	ZZHB-710	*Staphylea bumalda*	-	-	-	MF991905	[31]
*A. tenuissima*	15-245	*Aronia melanocarpa*	LC134324	LC134319	LC136865	-	[32]
	QHT3	*Begonia semperflorens*	MN264615	MN977123	MN256108	-	[33]
	KAC-3	*Actinidia chinensis*	-	-	-	MN395834	[34]
	GS-ATA2	*Solanum tuberosum*	-	-	-	JX495167	[35]
*A. gaisen*	CBS 118488	*Pyrus pyrifolia*	KP124427	KP124278	KP125206	-	[36]
	CBS 632.93	*Pyrus pyrifolia*	KC584197	KC584116	KC584658	-	[36]
*A. alstroemeriae*	CBS 118808	*Alstroemeria* sp.	KP124296	KP124153	KP125071	-	[37]
*A. betae-kenyensis*	CBS 118810	*Beta vulgaris* var. *cicla*	KP124419	KP124270	KP125197	-	[36]
*A. eichhorniae*	CBS 119778	*Eichhornia crassipes*	KP124426	KP124277	KP125205	-	[37]
*A. burnsii*	CBS 118817	*Tinospora cordifolia*	KP124424	KP124274	KP125202	-	[36]
*A. gossypina*	CBS 102597	*Minneola tangelo*	KP124432	KP124281	KP125211	-	[36]
*A. iridiaustralis*	CBS 118404	*Iris* sp.	KP124434	KP124283	KP125213	-	[37]
*A. jacinthicola*	CBS 878.95	*Arachis hypogaea*	KP124437	KP124286	KP125216	-	[36]
*A. longipes*	CBS 121332	*Nicotiana tabacum*	KP124443	KP124292	KP125222	-	[38]
*A. tomato*	CBS 103.30	*Solanum lycopersicum*	KP124445	KP124294	KP125224	-	[36]
*A. daucifolii*	CBS 118812	*Daucus carota*	KC584193	KC584112	KC584652	-	[37]
*A. calendulae*	CBS 224.76	*Calendula officinalis*	KJ718127	KJ717977	KJ718475	-	[29]
*A. nitrimali*	CBS 109163	Solanum viarum	KJ718201	JQ646358	KJ718547	-	[29]
*A. dichondrae*	CBS 199.74	Dichondra repens	KJ718166	JQ646357	KJ718514	-	[29]
*A. agripestis*	CBS 577.94	Euphorbia esula	KJ718099	JQ646356	KJ718448	-	[29]
*A. acalyphicola*	CBS 541.94	Acalypha indica	KJ718097	JQ646355	KJ718446	-	[29]
*A. tropica*	CBS 631.93	*Passiflora edulis*	KJ718261	JQ646354	KJ718607	-	[29]
*A. cirsinoxia*	CBS 113261	Cirsium arvense	KJ718143	KJ717993	KJ718491	-	[39]
*A. henanensis*	JZB3180070	*-*	MW793897	MW818009	MW818083	-	-
*A. guizhouensis*	JZB3180067	*-*	MW793894	MW818006	MW818080	-	-
*A. linariae*	NB242	*Solanum tuberosum*	KT968774	KR911762	KT937248	-	[40]
*A. solani*	NB266	*-*	KT968777	KR911753	KT937258	-	-
*A. tenuissima*	AvAt-1	*A. venetum*	OR602905	OR603956	OR672518	OR603950	In this study
	AvAt-2	*A. venetum*	OR602906	OR603957	OR672519	OR603951	In this study
	AvAt-3	*A. venetum*	OR602907	OR603958	OR672520	OR603952	In this study
	PhAt-1	*P. hendersonni*	OR602908	OR603959	OR672521	OR603953	In this study
	PhAt-2	*P. hendersonni*	OR602909	OR603960	OR672522	OR603954	In this study
	PhAt-3	*P. hendersonni*	OR6029010	OR603961	OR672523	OR603955	In this study
*Fusarium solani*	F174	*Cucurbita maxima*	-	-	-	MK497772.1	[41]

**Table 3:** The toxicity of hymexazol and ZSM fungicides on AvAt and PhAt strains.

		AvAt-2			PhAt-3	
Fungicides	Regression equation	Correlation coefficient	EC50/ mg/L	Regression equation	Correlation coefficient	EC_50_/ mg/L
Hymexazol	y = 2.7368x − 1.4447	0.9894	226.38	y = 2.3167x − 0.053	0.9925	151.75
ZSM	y = 0.5275x + 3.3771	0.7148	1192.86	y = 0.4850x + 1.8790	0.7435	987.96

**Table 4:** The inhibition ratio (%) of hymexazol and ZSM at different concentrations against *A. tenuissima*.

Fungicides	The Concentration of Fungicides	AvAt-2	PhAt-3
	58	58.35 ± 0.25 E b	62.42 ± 0.17 E a
	117	61.68 ± 1.66 D b	71.53 ± 0.53 D a
hymexazol	233	75.40 ± 0.80 C b	81.53 ± 0.43 C a
	467	88.10 ± 0.52 B b	94.08 ± 0.53 B a
	933	98.18 ± 0.23 A a	98.40 ± 0.00 A a
	17	12.00 ± 0.17 C b	20.00 ± 2.16 B a
	33	11.28 ± 0.98 C b	18.33 ± 4.14 B a
ZSM	67	11.75 ± 0.17 C b	24.18 ± 2.60 B a
	133	17.58 ± 0.34 B b	24.18 ± 2.60 B a
	267	37.43 ± 0.36 A b	43.73 ± 2.09 A a

Notes: Different capital letters indicate significant differences within the same columns of each fungicide at *p* < 0.05; different lowercase letters indicate significant differences within the same rows at *p* < 0.05.

## Data Availability

Not applicable.

## References

[1] Berljand S.S. (1950). Agro-Technology of Kendir.

[2] Xie W., Zhang X., Wang T., Hu J. (2012). Botany, traditional uses, phytochemistry and pharmacology of *Apocynum venetum* L. (Luobuma): A review. J. Ethnopharmacol..

[3] Jiang Y., Li B.T. (1977). China Botanical Records.

[4] Gao P., Duan T.Y., Christensen M.J., Nan Z.B., Liu Q.T., Meng F.J., Huang J.F. (2018). The occurrence of rust disease, and biochemical and physiological responses on *Apocynum venetum* plants grown at four soil water contents, following inoculation with *Melampsora apocyni*. Eur. J. Plant Pathol..

[5] Gao P., Duan T.Y., Nan Z.B., Christensen M.J., Liu Q.T., Meng F.J., Huang J.F. (2018). The influence of irrigation frequency on the occurrence of rust disease (*Melampsora apocyni*) and determination of the optimum irrigation regime in organic *Apocynum venetum* production. Agric. Water Manag..

[6] Gao P., Duan T.Y., Nan Z.B., O’Connor P.J. (2014). First report of *Septoria apocyni* causing spot blight on the species of *Apocynum venetum* and *Poacynum pictum* in China. Plant Dis..

[7] Gao P., Duan T.Y., Nan Z.B. (2017). *Alternaria catharanthicola* causes black leaf spot of *Apocynum venetum* in China. Plant Dis..

[8] Lan Y.R., Li T., Duan T.Y., Gao P. (2019). Effects of pappus removal and low-temperature short-term storage on interspecific and intraspecific variation in seed germination of Luobuma. Seed Sci. Technol..

[9] Lan Y.R., Duan T.Y. (2022). Characterization of *Boeremia exigua* causing stem necrotic lesions on Luobuma in northwest China. Sci. Rep..

[10] Pitt J.I., Hocking A.D. (1997). Fungi and Food Spoilage.

[11] Zheng H.H., Zhao J., Wang T.Y., Wu X.H. (2015). Characterization of *Alternaria* species associated with potato foliar diseases in China. Plant Pathol..

[12] Wang T., Zhao J., Sun P., Wu X. (2014). Characterization of *Alternaria* species associated with leaf blight of sunflower in China. Eur. J. Plant Pathol..

[13] Hong S.G., Cramer R.A., Lawrence C.B., Pryor B.M. (2005). *Alt a1* allergen homologs from *Alternaria* and related taxa: Analysis of phylogenetic content and secondary structure. Fungal Genet. Biol..

[14] Myresiotis C.K., Karaoglanidis G.S., Vryzas Z., Papadopoulou-Mourkidou Z. (2011). Evaluation of plant-growth-promoting rhizobacteria, acibenzolar-S-methyl and hymexazol for integrated control of *Fusarium* crown and root rot on tomato. Pest Manag. Sci..

[15] Payne P.A., Williams G.E. (1990). Hymexazol treatment of sugar-beet seed to control seedling disease caused by *Pythium* spp. and *Aphanomyces cochlioides*. Crop Prot..

[16] Tian Y.L., Jiang X.L., Ji J.H., Zhu C.X. (2010). Development and optimization of the chemically defined medium composition for ZSM production by *Streptomyces lavendulae* var. hainanensisn. Chin. J. Antibiot..

[17] Wang J.J., Chen T.X., Wei X.K., Kamran M., White J.F., Zhao G.Q., Li C.J. (2023). Evaluation of different antimicrobial agents for laboratory and field against *Pantoea agglomerans*, the causative agent of bacterial leaf blight disease on oat (*Avena sativa*). Plant Pathol..

[18] Song X.M., Zhu X.Y., Li T., Liang C., Zhang M., Shao Y., Tao J., Sun R.F. (2019). Dehydrozingerone inspired discovery of potential broad-spectrum fungicidal agents as ergosterol biosynthesis inhibitors. J. Agric. Food Chem..

[19] Wang Q., Zhang C., Long Y., Wu X., Su Y., Lei Y., Ai Q. (2021). Bioactivity and control efficacy of the novel antibiotic tetramycin against various kiwifruit diseases. Antibiotics.

[20] Gao P., Nan Z.B., Christensen M.J., Barbetti M.J., Duan T.Y., Liu Q.T., Meng F.J., Huang J.F. (2019). Factors influencing rust (*Melampsora apocyni*) intensity on cultivated and wild *Apocynum venetum* in Altay Prefecture, China. Phytopathology.

[21] Simmons E.G. (2007). Alternaria: An Identification Manual.

[22] Tao Y., Zeng F., Ho H., Wei J., Wu Y., Yang L., He Y. (2011). *Pythium vexans* causing stem rot of *Dendrobium* in Yunnan province, China. J. Phytopathol..

[23] White T.J., Bruns T., Lee S., Taylor J., Innis M.A., Gelfand D.H., Spinsky T.J., White T.J. (1990). Amplification and direct sequencing of fungal ribosomal RNA genes for phylogenetics. PCR Protocols: A Guide to Methods and Applications.

[24] Berbee M.L., Pirseyedi M., Hubbard S. (1999). *Cochliobolus* phylogenetics and the origin of known, highly virulent pathogens, inferred from ITS and glyceraldehyde-3-phosphate dehydrogenase gene sequences. Mycologia.

[25] Jacobs K., Bergdahl D.R., Wingfield M.J., Halik S., Seifert K.A., Bright D.E., Wingfield B.D. (2004). *Leptographium wingfieldii* introduced into North America and found associated with exotic *Tomicus pimiperda* and native bark beetles. Mycol. Res..

[26] Kumar S., Stecher G., Tamura K. (2016). MEGA7: Molecular evolutionary genetics analysis version 7.0 for bigger datasets. Mol. Biol. Evol..

[27] Kgatle M.G., Truter M., Ramusi T.M., Flett B., Aveling T.A.S. (2018). *Alternaria alternata*, the causal agent of leaf blight of sunflower in South Africa. Eur. J. Plant Pathol..

[28] Wei X.K., Xue L.H., Li C.J. (2021). The first report of leaf spot caused by *Alternaria alternata* on Italian ryegrass (*Lolium multiflorum*) in China. Plant Dis..

[29] Lawrence D.P., Gannibal P.B., Peever T.L., Pryor B.M. (2013). The sections of *Alternaria*: Formalizing species-group concepts. Mycologia.

[30] Gilardi G., Matic S., Gullino M.L., Garibaldi A. (2019). First report of *Alternaria alternata* causing leaf spot on Spinach (*Spinacia oleracea*) in Italy. Plant Dis..

[31] Wei M., Chen J.M., Fu B.Z., Li G.Y., Wang X.S. (2018). First report of brown leaf blight of Shenguyou (*Staphylea bumalda*) caused by *Alternaria alternata* in China. Plant Dis..

[32] Wee J.I., Park J.H., Back C.G., You Y.H., Chang T. (2016). First report of leaf spot caused by *Alternaria tenuissima* on black chokeberry (*Aronia melanocarpa*) in Korea. Mycobiology.

[33] Zhang X., Jiang B., Wang R., Li Y., Sun L. (2020). Occurrence of leaf spot of *Begonia semperflorens* caused by *Alternaria tenuissima* in China. Plant Dis..

[34] Ma J., Li H., Wang X., Guo M. (2020). *Alternaria tenuissima* causing fruit scab disease on *Actinidia chinensis* in Anhui province, China. Plant Dis..

[35] Zheng H.H., Wu X.H. (2013). First report of Alternaria blight of potato caused by *Alternaria tenuissima* in China. Plant Dis..

[36] Carbone I., Kohn L.M. (1999). A method for designing primer sets for speciation studies in flamentous ascomycetes. Mycologia.

[37] Gou Y.N., Aung S.L.L., Htun A.A., Huang C.X., Deng J.X. (2022). *Alternaria* species in section *Alternaria* associated with Iris plants in China. Front. Plant Microbiol..

[38] Woudenberg J.H., Seidl M.F., Groenewald J.Z., de Vries M., Stielow J.B., Thomma B.P., Crous P.W. (2015). Alternaria section *Alternaria*: Species, formae speciales or pathotypes?. Stud. Mycol..

[39] Cai Z.Y., Liu Y.X., Shi Y.P., Dai L.M., Li L.L., Mu H.J., Lv M.L., Liu X.Y. (2019). *Alternaria yunnanensis* sp. nov., a new *Alternaria* species causing foliage spot of Rubber tree in China. Mycobiology.

[40] Bessadat N., Hamon B., Henni D.E., Simoneau P. (2016). First report of tomato early blight caused by *Alternaria grandis* in Algeria. Plant Dis..

[41] Moumni M., Allagui M.B., Mancini V., Murolo S., Tarchoun N., Romanazzi G. (2020). Morphological and molecular identification of seedborne fungi in Squash (*Cucurbita maxima*, *Cucurbita moschata*). Plant Dis..

[42] Li Y., Zhang D., Xu W., Wu Z., Cao A. (2011). *Alternaria tenuissima* causing leaf spot and fruit rot on pepper (*Capsicum annuum*): First report in China. New Dis. Rep..

[43] Fu R., Chen C., Wang J., Ke Y., Lu D. (2019). Identification of *Alternaria tenuissima* causing brown leaf spot on *Paris polyphylla* var. *chinensis* in China. J. Phytopathol..

[44] Nasehi A., Kadir J.B., Abidin M., Wong M.Y., Mahmodi F. (2012). First report of *Alternaria tenuissima* causing leaf spot on eggplant in Malaysia. Plant Dis..

[45] Lee J.H., Kim D.S., Cho H.J., Gang G.H., Kwak Y.S. (2013). First report of leaf spot in *Farfugium japonicum* caused by *Alternaria tenuissima* in Korea. Plant Dis..

[46] Bensassi F., Zid M., Rhouma A., Bacha H., Hajlaoui M.R. (2009). First report of *Alternaria* species associated with black point of wheat in Tunisia. Ann. Microbiol..

[47] Lin Q., Sun X.T., He H., Yang D.X. (2016). First report of leaf spot caused by *Alternaria tenuissima* on *Avicennia marina* in China. Plant Dis..

[48] Song X.B., Cui Y.P., Peng A.T., Ling J.F., Chen X. (2020). First report of brown spot disease in *Psidium guajava* caused by *Alternaria tenuissima* in China. J. Plant Pathol..

[49] Gao P., Nan Z.B., Wu Y.N., Liu Q.T., Meng F.J., Xiao Z.C., Duan T.Y. (2015). Characteristics photosynthetic physiology and growth with 8 Luobuma ecotypes in the *Apocynum* and *Poacynum* from Altay of Xinjiang, China. Acta Botonica Boreali Occident. Sinaca.

[50] Rotem J. (1994). The genus *Alternaria*: Biology, epidemiology, and pathogenicity. Biol. Epidemiol. Pathog..

[51] Andrew M., Peever T.L., Pryor B.M. (2009). An expanded multilocus phylogeny does not resolve morphological species within the small-spored *Alternaria* species complex. Mycologia.

[52] Tymon L.S., Peever T.L., Johnson D.A. (2016). Identification and enumeration of small-spored *Alternaria* species associated with potato in the US northwest. Plant Dis..

[53] Siciliano I., Gilardi G., Ortu G., Gisi U., Gullino M.L., Garibaldi A. (2017). Identification and characterization of *Alternaria* species causing leaf spot on cabbage, cauliflower, wild and cultivated rocket by using molecular and morphological features and mycotoxin production. Eur. J. Plant Pathol..

[54] Bai Y.B., Zhang A.L., Tang J.J., Gao J.M. (2013). Synthesis and antifungal activity of 2-Chloromethyl-1H-benzimidazole derivatives against phytopathogenic fungi in vitro. J. Agric. Food Chem..

[55] Tang H.Y., Zhang Q., Li H., Gao J.M. (2015). Antimicrobial and allelopathic metabolites produced by *Penicillium brasilianum*. Nat. Prod. Res..

[56] Wang Y., Huang D., Cheng Y.X. (2023). Structural optimization, fungicidal activities evaluation, DFT study and structure-activity relationship of dopamine derivatives with benzothiazole fragment from *Polyrhachis dives*. Chem. Biodivers..

[57] Wang S., Bao L., Wang W., Song D., Wang J., Cao X. (2018). Heterocyclic pyrrolizinone and indolizinones derived from natural lactam as potential antifungal agents. Fitoterapia.

[58] Ren Y., Li D., Jiang S., Wang Y., Tang Q., Huang H., Wang D., Song B., Chen Z. (2021). Integration of transcriptomic and proteomic data reveals the possible action mechanism of the antimicrobial ZSM against *Didymella segeticola*, the causal agent of tea leaf spot. Phytopathology.

[59] Jiang X., Zhu C., Ji J., Sun D., Wei G., Tian Y. (2003). Mechanism of zhongshengmycin in control of *Xanthomonas oryzae* pv. *oryzae*. Chin. J. Biol. Control.

[60] Wang Q., Lin M., Shen P., Guan Y. (2021). Elevation of fatty acid biosynthesis metabolism contributes to Zhongshengmycin resistance in *Xanthomonas oryzae*. Antibiotics.

[61] Tao H., Wang H., Huang S.X., Zhang Y., Zhang Z.H., Liu W., Shi N.X., Zhu F., Ji Z.L., Chen X.R. (2020). Identification and characterization of *Diaporthe eres* causing leaf blight disease on the medicinal herb *Polygonatum sibiricum*. J. Gen. Plant Pathol..

